# Dissecting the Neural Circuitry for Pain Modulation and Chronic Pain: Insights from Optogenetics

**DOI:** 10.1007/s12264-022-00835-8

**Published:** 2022-03-05

**Authors:** Fang Guo, Yu Du, Feng-Hui Qu, Shi-Da Lin, Zhong Chen, Shi-Hong Zhang

**Affiliations:** 1 Department of Pharmacology and Department of Anesthesiology of the Second Affiliated Hospital, Zhejiang University School of Medicine, Hangzhou, 310058 China; 2 Key Laboratory of Neuropharmacology and Translational Medicine of Zhejiang Province, College of Pharmaceutical Sciences, Zhejiang Chinese Medical University, Hangzhou, 310053 China

**Keywords:** Optogenetics, Pain transmission, Pain modulation, Chronic pain, Neural circuits

## Abstract

Pain is an unpleasant sensory and emotional experience associated with, or resembling that associated with, actual or potential tissue damage. The processing of pain involves complicated modulation at the levels of the periphery, spinal cord, and brain. The pathogenesis of chronic pain is still not fully understood, which makes the clinical treatment challenging. Optogenetics, which combines optical and genetic technologies, can precisely intervene in the activity of specific groups of neurons and elements of the related circuits. Taking advantage of optogenetics, researchers have achieved a body of new findings that shed light on the cellular and circuit mechanisms of pain transmission, pain modulation, and chronic pain both in the periphery and the central nervous system. In this review, we summarize recent findings in pain research using optogenetic approaches and discuss their significance in understanding the pathogenesis of chronic pain.

## Introduction

According to the International Association for the Study of Pain (IASP), pain is an unpleasant sensory and emotional experience associated with, or resembling that associated with, actual or potential tissue damage [1]. The processing of pain engages a wide variety of structures across the nervous system and involves complex regulation. Under physiological conditions, pain caused by actual or potential tissue injury is sudden in onset and time-limited. This form of pain is termed acute pain [2]. It is a warning signal that alerts the body to avoid noxious stimulation and hence protects the body from further damage. On the other hand, chronic pain persists or recurs for >3 months [3]. Epidemiological data show that 33% of adults and 56% of the elderly in developing countries suffer from chronic pain, such as joint pain, low back pain, and widespread pain [4]. In these patients, pain can be evoked or exacerbated by external innocuous or noxious stimulation, respectively, or occurs spontaneously when there is no obvious inducement. In such cases, pain loses its significance for protection and turns into a kind of disease. Both acute and chronic pain causes much suffering, seriously affects the quality of life of patients, and is a huge burden to both families and society [4]. At present, pain is mainly managed with medications that have many shortcomings such as poor effectiveness and a variety of adverse reactions [5]. A better understanding of pain is urgently required.

The sensory transmission of pain starts from the activation of peripheral nociceptors and the generation of nociceptive action potentials evoked by noxious stimulation, by which the signals are transmitted along primary afferent fibers to the second-order sensory neurons in the spinal dorsal horn or the brain stem. After being integrated, these signals are transmitted to multiple brain regions related to pain. As the first station of pain transmission, nociceptors are functionally diverse and specific. Noxious heat and mechanical stimulation are detected by different nociceptors [6, 7]. Nociceptors that are involved in different forms of chronic pain, such as inflammatory pain and neuropathic pain, are also different [7–9]. The spinal dorsal horn is not only the relay station of sensory transmission, but also the primary center for integrating sensory information. Sensory neurons in the dorsal horn are regulated by local interneurons and glial cells, sensory afferents, and descending projections from supraspinal structures. Under pathological conditions, the regulatory function can be compromised, which may lead to increased neuronal excitability, a phenomenon termed central sensitization [10]. Previous studies have revealed that several brain regions, such as the parabrachial nucleus (PBN), thalamus, sensory cortex, anterior cingulate cortex (ACC), prefrontal cortex (PFC), nucleus accumbens (NAc), and amygdala are involved in pain perception, emotional responses, and other dimensions of pain. The roles of these nuclei in pain modulation and chronic pain are not fully understood and are emerging as hot issues in the field of pain research.

Optogenetics is a novel biotechnology combining optical and genetic technology that enables precise intervention in a specific group of neurons or a circuit element by light stimulation [11]. When applied to living animals, optogenetics is a useful tool to investigate the neural circuits related to a certain behavior. The working principle of optogenetics is to transfer an exogenous opsin (photosensitive protein) gene from microorganisms into specific type of mammalian cells with viral vectors. Opsin, the key factor in optogenetics, is then expressed on the cell membrane and its conformation changes in response to proper light stimulation with rapid and frequent reversals (the temporal precision of response can reach the millisecond level). Opsins are usually ion channel proteins, ion pumps, and G protein-coupled receptors (GPCRs). The commonly used opsins in neuroscience research as well as their characteristics are briefly summarized in Table 1. These opsins are engineered or modified from the wild-type to improve the on-off kinetics and light sensitivity, making them more suitable for animal neuroscience research. With the advantages of high specificity, sensitivity, and precision, all of which had been impossible in the past with classical techniques such as pharmacological intervention and electrical stimulation, optogenetics provides strong technical support for elucidating the neural circuitry of pain transmission and modulation as well as the pathogenesis of chronic pain. In this review, we summarize recent studies on pain that take advantage of optogenetic approaches and discuss their significance in understanding pain modulation and chronic pain.

**Table 1:** Commonly-used Opsins in Neuroscience Research

Function	Opsin	Protein nature	Activation wavelength (nm)	Feature
Excitatory	Channelrhodopsin (ChR) 2	Nonspecific cation channel	470	Slow kinetics
	ChETA	ChR2 variant	470	Faster kinetics
	VChR1	*Volvox* ChR1	589	Red-shifted
	oChIEF	Chimeric opsin of ChR1 and ChR2	450–470	Reliable response to high-frequency stimulation
	ReaChR	Red-activatable ChR	590–630	Red-shifted, more light sensitive
	Chronos	ChR	500–530	More light sensitive, faster kinetics
	Chrimson	ChR	590–600	Red-shifted
	ChroME	Chronos variant	490	Ultrafast and highly potent
	C1V1	Chimeric opsin of ChR1 and VChR1	540–560	Red-shifted, suitable for two-photo optogenetics
	SFOs	Step function opsins	470	Deactivated by 542 nm green light
	SSFOs	Stabilized step-function ChR2	470	Deactivated by 590 nm yellow light
Inhibitory	eNpHR	Engineered halorhodopsin, chloride pump	589	More light sensitive
	Archaerhodopsin (ArchT)	Enhanced proton pump	566	More potent
	Mac	Proton pump	540	
	ChloCs	Chloride-conducting ChR	465	Increased light sensitivity
	iC1C2	Chloride-conducting ChR	475	More light sensitive
	Jaws	Cruxhalorhodopsin, chloride-conducting	632	Red-shifted, highly light sensitive
	GtACR1	Anion-conducting ChR	515	Fast kinetics, high potency

## Application and Findings of Optogenetics in the Study of Pain

### Primary Sensory Neurons

Although peripheral nociceptive signals are known to be transmitted through δ (thinly myelinated) and C (unmyelinated) afferent fibers, the roles of primary sensory neurons in encoding and transmitting pain sensation and chronic pain are not fully understood. Taking advantage of optogenetics, a few studies have revealed some new fiber connections and functions of specific types of primary sensory neurons as well as their roles in nociception and chronic pain. Aβ fibers that normally encode innocuous mechanical stimulation have been proposed to play a role in neuropathic pain, but whether their activation indeed evokes pain after nerve injury remained uncertain. Using a transgenic rat line in which ChR2 is selectively expressed in Aβ fibers, Tashima *et al.* found that the selective activation of Aβ fibers after peripheral nerve injury elicits pain-like behaviors and aversion, together with excitation of neurons in the superficial dorsal horn and amygdala [12]. This study provides direct evidence for a contribution of Aβ fibers to neuropathic pain.

A subset of the mas-related genes (Mrgs), namely sensory neuron-specific Mrg GPCRs, are selectively expressed in the dorsal root ganglia (DRG) and can be used as a marker of non-peptidergic neurons [13, 14]. In spinal cord slices, optogenetic activation of the terminals of neurons expressing Mrgprd (an Mrg subtype) evokes excitatory postsynaptic potentials in 50% of dorsal horn lamina II neurons. These fibers form monosynaptic connections to most known classes of dorsal horn neuron, ruling out the possibility that Mrgprd-positive neurons innervate a dedicated class of these neurons [15]. The activation of these neurons is not normally aversive, but becomes so after peripheral nerve injury. This shift may be due to the recruitment of a new polysynaptic connection with dorsal horn neurons projecting to the PBN [16]. This study indicates that non-peptidergic primary afferents contribute to neuropathic pain, possibly by changing connections with second-order sensory neurons. On the other hand, transient receptor potential vanilloid-1 is restricted to a specific subset of peptidergic primary sensory neurons. Selective epineural activation of peripheral axons of these neurons evokes pain-like behaviors [17], confirming their nociceptive nature. Tachykinin 1 (Tac1) is also expressed in peptidergic nociceptors. Optical activation of these nociceptors in the skin results in nocifensive behaviors and the activation of neurons in the superficial laminae of the dorsal horn [18]. These studies verify the classical concept that peptidergic primary afferents are nociceptive. In addition, by injecting virus into individual DRGs and implanting light-emitting diodes nearby, the focal DRG-specific control of visceral and/or somatic afferents can be precisely regulated by light stimulation with different parameters. This technique has been used to investigate the role of DRG neurons in pain sensation from specific visceral organs *in vivo* [19], and will help to further understand referred pain.

Nav1.8, the voltage-gated sodium channel expressed in primary sensory neurons, participates in the generation and transmission of action potentials, and is involved in inflammatory pain and neuropathic pain [20]. In Nav1.8-ChR2 transgenic mice constructed by knocking the ChR2 gene into Nav1.8-cre mice, remote or epidural blue light illumination induces the activation of sensory neurons and central sensitization in the spinal cord, as well as mechanical hypersensitivity, avoidance behavior, and conditioned place aversion in animals [21, 22]. By contrast, in Nav1.8-Arch mice, selective inhibition of Nav1.8-expressing afferents by acute or prolonged epidural or transdermal illumination induces analgesia under physiological or pathological (inflammatory and neuropathic) conditions [22, 23]. These studies support the hypothesis that Nav1.8-positive afferents play key roles in pain transmission and chronic pain.

Tropomyosin-related kinase B (TrkB) receptors are expressed in some DRG and dorsal horn neurons, especially in the superficial laminae. After release in the dorsal horn, brain-derived neurotropic factor (BDNF) binds to TrkB receptors and activates tyrosinase activity, which then triggers a cascade of signal conduction that participates in pain transmission and modulation. Dhanapani *et al.* [24] found that the expression of BDNF and TrkB receptors is increased under the conditions of nerve injury and peripheral inflammation. Optogenetic activation of TrkB-expressing neurons promotes, while depletion of these neurons with diphtheria toxin abolishes, the mechanical allodynia in mice after sciatic nerve injury. These results demonstrate the necessity and sufficiency of TrkB-expressing neurons for neuropathic allodynia, and indicate that ligand-guided laser ablation of TrkB-positive neurons is expected to be a new approach to relieving neuropathic pain. Along with the discovery of new markers for different types of primary sensory neurons, it can be expected that optogenetics will facilitate the understanding of their roles in pain transmission and chronic pain.

### Spinal Cord

The spinal dorsal horn is the primary center for the transmission and integration of nociceptive signals. With the identification of markers for different linage neurons in the dorsal horn, it has been revealed that these neurons are involved in different modalities of pain [25–28]. Along with the improvement of devices and methodology, the application of optogenetics at the spinal level helps elucidate the cellular, molecular, and circuit mechanisms of pain transmission and integration with less tissue damage [22, 29]. Second-order sensory neurons expressing Tac1 receptor (Tacr1) and GPCR 83 respond to distinct types of stimulation and innervate distinct sets of neurons in the lateral PBN; optogenetic activation of their axon terminals in the PBN induces distinct escape behaviors and autonomic responses [30]. This study reveals two parallel ascending spinal pathways for pain. Local inhibitory interneurons and descending projections from supraspinal structures are closely involved in regulating the activity of second-order sensory neurons in the dorsal horn. Optical inhibition of γ-aminobutyric acid (GABA)-expressing interneurons in the dorsal horn induces mechanical but not thermal hyperalgesia, demonstrating that GABAergic modulation tonically inhibits mechanical nociception primarily and that mechanical and thermal nociception involve different modulatory mechanisms at the spinal level [22]. By contrast, calcium-binding protein calretinin-expressing interneurons relay sensory signals to superficial neurons projecting to the PBN. Photoactivation of these neurons induces mechanical allodynia and nocifensive behavior as well as conditioned place aversion. These effects are enhanced under neuropathic conditions [31, 32]. These studies together verify that the ascending information from second-order sensory neurons is tightly regulated by local interneurons, and the dysfunction of these local circuits contributes to pathological pain.

**Fig. 1:**
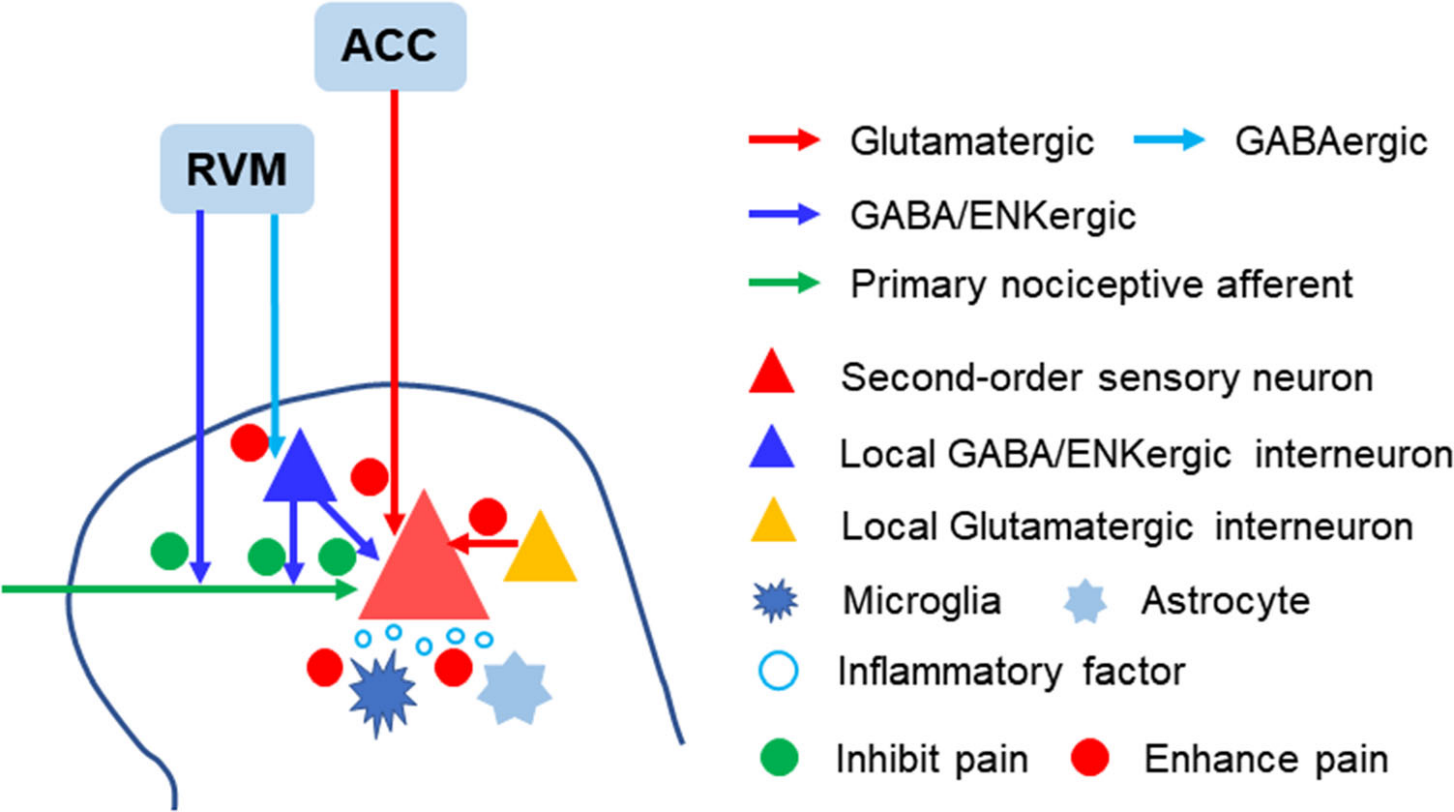
Circuits for the integration of nociceptive signals identified by optogenetics in the spinal cord. Local interneurons, either glutamatergic or GABA/ENKergic, enhance or inhibit pain by directly activating or inhibiting second-order sensory neurons in the dorsal horn. The RVM modulates the inputs from the central terminals of primary nociceptive afferents directly by presynaptic inhibition or by disynaptic activation through inhibiting local inhibitory interneurons. The ACC sends descending glutamatergic projections and directly enhances the excitatory neurotransmission of second-order sensory neurons. Microglia and astrocytes promote pain by releasing inflammatory factors. ACC, anterior cingulate cortex; ENKergic, enkephalinergic; RVM, rostroventral medulla.

Second-order sensory neurons and local interneurons, as well as the central terminals of primary sensory neurons, at the spinal level receive inputs from supraspinal structures. Previous studies have identified a few brainstem nuclei such as the rostroventral medulla (RVM), midbrain periaqueductal gray (PAG), dorsal raphe nucleus (DRN), locus coeruleus (LC), and reticular formation are the predominant components of the descending modulatory system. Zhang *et al.* [33] found that the downward projections of the RVM are mainly from dual GABAergic and enkephalinergic neurons. Selective activation of these projections induces depolarization at short latency in the dorsal roots, implying monosynaptic connections between RVM neurons and primary afferent neurons. Silencing or activating these RVM neurons substantially increases or decreases heat and mechanical nociception, confirming that GABA and enkephalin exert presynaptic inhibition on primary afferents. Another study revealed that GABAergic RVM neurons also innervate enkephalinergic/GABAergic interneurons in the dorsal horn that gate sensory inputs and control pain by presynaptic inhibition of primary sensory neurons. Optical inhibition of this circuit increases mechanical threshold, while its activation induces robust mechanical hypersensitivity, but neither manipulation affects heat nociception. These results indicate that the disynaptic inhibitory circuit from RVM descending projections to primary afferents facilitates mechanical rather thermal pain, supporting the concept that different modalities of pain involve different mechanisms [34]. Spinal cord-projecting RVM neurons identified by these two studies may either belong to different subgroups or send collateral rami to different targets, jointly functioning to maintain pain homeostasis. In addition, second-order sensory neurons receive direct innervation from the cortex, such as the ACC. Activation of spinal ACC projections potentiates spinal excitatory transmission and causes behavioral pain sensitization, while their inhibition has an analgesic effect [35]. Together, the results of these studies provide new insights into the integrative and modulatory system for pain at the spinal level (Fig. 1).

A large body of immunohistochemical and pharmacological evidence has accumulated supporting a key role of spinal neuroinflammation in neuropathic pain. A recent study demonstrates that optogenetic activation of spinal microglia is sufficient to trigger chronic pain phenotypes by increasing neuronal activity *via* interleukin-1 signaling [36]. On the other hand, photostimulation of ChR2-expressing spinal astrocytes also induces the production of proalgesic mediators and pain hypersensitivity [37]. These results provide direct evidence for the causative role of spinal neuroinflammation characterized by glial activation in pathological pain.

### Brain

**Fig. 2:**
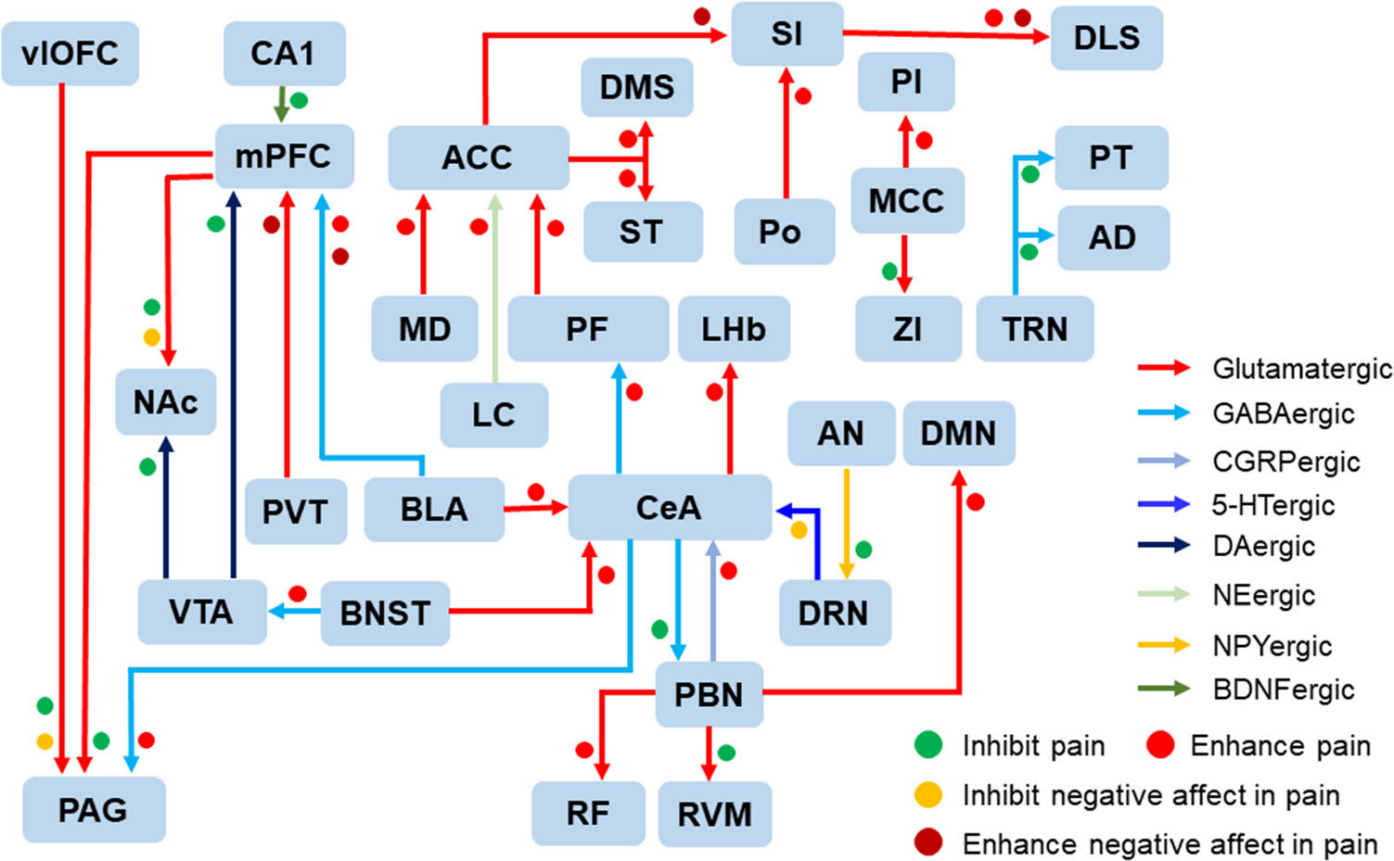
Neural circuits involved in pain modulation and chronic pain identified by optogenetics in the brain. ACC, anterior cingulate cortex; AD, anterodorsal thalamic nucleus; AN, arcuate nucleus; BLA, basal lateral amygdala; BNST, bed nucleus of the stria terminalis; CeA, central extended amygdala; DLS, dorsolateral striatum; DMS, dorsomedial striatum; DRN, dorsal raphe nucleus; CMN, central medial nucleus of the thalamus; LC, locus coeruleus; LHb, lateral habenula; MCC, midcingulate cortex; MD, mediodorsal thalamus; mPFC, medial prefrontal cortex; NAc, nucleus accumbens; PAG, periaqueductal gray; PBN, parabrachial nucleus; PF, parafascicular nucleus; PI, posterior insula; Po, posterior thalamic nucleus; PT, paratenial thalamic nucleus; PVT, paraventricular nucleus; SI, primary sensory cortex; ST, sensory thalamus; RF, reticular formation; RVM, rostroventral medulla; TRN, thalamic reticular nucleus; vlOFC, ventrolateral orbitofrontal cortex; VTA, ventral tegmental area; ZI, zona incerta.

Conventionally, nociceptive sensory information is transmitted upward from the spinal cord partly through the thalamus to the sensory cortex, processing information related to sensory characteristics, such as the location and intensity of stimulation, and partly through the brainstem and amygdala to the cingulate cortex, insular cortex, and other nuclei that are closely associated with the affective and cognitive aspects of pain such as aversion, fear, anxiety, and depression. Sensory inputs to the RVM and PAG either are transmitted upwards or activate the descending pain modulatory system. By combining optogenetics with imaging technology, researchers have found that cortical and subcortical structures form a complex neural network for the ascending transmission and descending modulation of pain, which also play important roles in the occurrence and maintenance of chronic pain. The circuits in the brain that have been shown to be involved in pain processing and modulation by optogenetics are schematized in Fig. 2.

#### Brain Stem

Nuclei in the brain stem involved in pain predominantly include the PBN, RVM, PAG, ventral tegmental area (VTA), and LC. Descending fibers from these nuclei to the dorsal horn are known to play a role in pain modulation (e.g., the GABAergic and enkephalinergic projections from the RVM noted above). Evidence from optogenetic studies has extended our understanding of the mechanisms of pain modulation and chronic pain.

The PBN is traditionally considered to be involved in both pain transmission and pain modulation. Optogenetic studies have revealed a highly refined function of the PBN. The nociceptive neurons in the PBN receive inputs from the spinal dorsal horn and trigeminal sensory nuclei, and interestingly, directly from trigeminal ganglion neurons [18, 38, 39]. The monosynaptic connection with the trigeminal nerve provides a neural substrate for heightened responses of PBN neurons and aversive behavior to noxious stimulation of the face [39]. These nociceptive PBN neurons project to many pain-, emotion- and instinct-related centers in the brain. It has been revealed that calcitonin gene-related peptide (CGRP)-positive neurons relay pain signals to the amygdala and mediate pain responses and threat memory [40]. On the other hand, Tac1-positive neurons relay nociceptive signals from the dorsal horn to the reticular formation to mediate escape responses to heat [18], while those expressing Tacr1 transmit signals to the central medial nuclei of the thalamus rather than the amygdala to mediate multimodal nociception [38]. The function of the PBN is modulated by other nuclei. Alhadeff *et al.* [41] reported that hunger-sensitive agouti-related protein-positive neurons in the arcuate nucleus that can be activated by hunger form synaptic connections with neurons in the lateral PBN. Selective activation of these projection terminals results in the attenuation of inflammatory pain but not of mechanical and thermal nociception, with the involvement of local neuropeptide Y (NPY) signaling. This study also revealed that the NPY system in the PBN participates in pain modulation in a modality-specific manner. Another study found that optogenetic activation of GABAergic neurons in the lateral PBN does not affect basal nociception, while inhibition of glutamatergic neurons reduces it, but both manipulations alleviate neuropathic pain after sciatic nerve injury. This indicates that a delicate balance between excitatory and inhibitory innervation of lateral PBN neurons plays an important role in neuropathic pain [42]. These studies together demonstrate that neuronal circuits both outside and inside the PNB are involved in pathological pain, underlining the key role of this nucleus.

The RVM, which contains three types of neurons based on their responses to noxious stimulation – on cells, off cells, and neutral cells – receives abundant inputs from the PBN. Optogenetic inhibition of projection terminals from the PBN decreases the spontaneous firing of on cells but increases that of off cells, indicating that the PBN participates in pain modulation *via* a circuit to pain modulatory cells in the RVM [43]. The postsynaptic currents evoked by photoactivation of PBN projections are blocked by a glutamate receptor antagonist but not a GABAa receptor antagonist in 3/4 of RVM neurons, while the responses of the remaining neurons are the opposite [43], indicating that the projection terminals of the PBN release either glutamate or GABA onto individual RVM neurons without concurrent release. This study reveals that the RVM mediates, at least in part, modulatory role of the PBN as well as the involved neurotransmitters.

Although descending pontospinal noradrenergic pathways are traditionally viewed as pain inhibitory, it seems that noradrenergic neurons in the LC can either inhibit or facilitate pain. It has been reported that optical activation of LC neurons evokes bidirectional changes in thermal nociception in rats. Anatomical analysis reveals that activation of ventral LC neurons is responsible for the anti-nociceptive effect, while the pro-nociceptive effect may be mediated by distinct subgroups of LC neurons that connect with other brain regions [44]. In support of this, Koga *et al.* reported that photoactivation of norepinephrinergic terminals in the ACC enhances excitatory transmission and induces scratching and behavioral sensitization for mechanical stimulation, suggesting that LC norepinephrinergic neurons facilitate pain and itch perception by potentiating glutamatergic transmissions in the ACC [45]. Moreover, the LC has been proposed to be a chronic pain generator [46], which has not been demonstrated by the optogenetic approach, although there are several lines of pharmacological evidence.

The VTA is rich in dopaminergic neurons. Optogenetic activation of these neurons or their projection terminals in the NAc attenuates pain-like behaviors in the mouse model of neuropathic pain or cancer pain [47]. Besides, phasic activation of the DA inputs into the mPFC reduces neuropathic mechanical hypersensitivity, possibly by increasing the activity of PAG-projecting neurons [48]. Interestingly, it has been shown that after peripheral nerve injury, VTA neurons projecting to the NAc increase the spontaneous firing frequency, which contributes to thermal hyperalgesia, whereas those projecting to the mPFC decrease activity, which is not related to thermal hyperalgesia [49]. These studies demonstrate the differential modulatory functions of these dopaminergic pathways. The VTA receives inhibitory inputs from the dorsolateral bed nucleus of the stria terminalis. This innervation is strengthened in chronic pain states following nerve injury, resulting in tonic inhibition of dopaminergic function in the midbrain limbic system [50]. These studies indicate that the hypoactivity of dopaminergic neurons in the VTA underlies pathological pain and its restoration may serve as a therapeutic strategy.

#### Diencephalon

The thalamic nuclei are classically regarded as relay centers of sensory information transmitted from the spinal dorsal horn and the brainstem to the cortex. Studies have revealed that ascending sensory inputs and descending modulatory inputs from the cortex converge in the thalamus and mutually influence each other [51]. The concept that the thalamus participates in pain modulation is supported by optogenetic studies. It has been found that the glutamatergic circuit from the posterior thalamic nucleus to the primary somatosensory cortex (S1) mediates allodynia from tissue injury, whereas the GABAergic circuit from the parafascicular nucleus to the ACC mediates allodynia associated with a depression-like state [52], indicating that different thalamocortical circuits underlie pathological pain with different etiologies. The thalamic reticular nucleus (TRN) participates in the transmission of nociceptive signals. Optogenetic activation of parvalbumin (PV)-positive GABAergic neurons in the rostro-dorsal sector of the TRN facilitates mechanical and thermal nociception through the circuits to the anterodorsal and paratenial thalamic nuclei [53]. Optogenetic activation of glutamatergic neurons in the posterior portion of the thalamic paraventricular nucleus (PVT) that send projections to the central extended amygdala (CeA) induces mechanical hypersensitivity in naïve rats. This pain facilitation is mediated by projections from the CeA to the ventrolateral PAG (vlPAG). The activity of this circuit is increased after peripheral nerve injury, whereas lesion or inhibition of the circuit alleviates mechanical allodynia [54]. This study identifies a new pain-facilitating circuit that contributes to neuropathic pain and provides a potential target for pain intervention.

The lateral habenula (LHb) is an area known to be involved in aversion and depression. Although the majority of LHb neurons are excited by noxious stimuli, until recently evidence for its role in pain modulation and neuropathic pain has been emerging. We recently reported that optogenetic manipulation of glutamatergic LHb neurons does not affect mechanical sensitivity, indicating a negligible role in modulating mechanical nociception. However, these neurons become hyperactive after peripheral nerve injury. More importantly, both optogenetic inhibition and electrical stimulation of the LHb at low frequency produce analgesic effect [55]. These results demonstrate that a hyperactive LHb promotes neuropathic pain, and inhibition of LHb activity may serve as a potential treatment strategy.

#### Basal Ganglia

The NAc receives afferents from the PFC. Imaging studies suggest that this connection is altered in chronic pain [56]. Selective activation of the projection terminals in the NAc of neurons in the prelimbic cortex (PrL), a subregion of the mPFC, reduces not only acute pain and aversion, but also the sensory and affective symptoms of neuropathic pain, demonstrating that the PFC–NAc circuit serves as a potential target for the management of neuropathic pain [57, 58]. Massaly *et al.* revealed that the elevated activity of κ opioid receptors in a section of the NAc shell underlies the reduced motivational state in inflammatory pain. Selective activation of the local dynorphin-containing neurons is sufficient to drive κ receptor-dependent negative affective states and aversive behavior [59]. This study provides evidence that pain-induced adaptations in the κ opioid system within the NAc shell are a potential target for therapeutic intervention in pain-induced affective disorders.

The amygdala is involved not only in processing negative affect associated with pain such as fear, anxiety, and aversion, but also in pain modulation and chronic pain. The CeA, the major output nucleus of the amygdala, is considered to be the nociceptive amygdala and has extensive connections to the forebrain and the brainstem [60]. The CGRP-signaling pathway in the CeA may mediate the role of CGRP-positive terminals from the PBN in the affective aspect of pain [40]. GABAergic neurons in the CeA project to glutamatergic neurons in the parafascicular nucleus that relay directly to neurons in the secondary sensory cortex. The activity of this inhibitory pathway is enhanced in mice with comorbid pain in depression, while reversing this change using an optogenetic approach alleviates pain [61]. Another group of neurons in the CeA, somatostatin-expressing neurons, receive inhibitory innervation from the DRN *via* 5-HT_1A_ receptors and send abundant projections to the LHb [62]. Nerve injury reduces the inhibitory strength of DRN neurons and hence increases the activity of LHb-projecting CeA neurons and enhances glutamatergic neurotransmission, which may explain the hyperactive state of the LHb after nerve injury [55]. Optogenetic reversal of these changes ameliorates both neuropathic pain and the concurrent depression-like behaviors [62]. This study reveals a new molecular and circuitry mechanism for the occurrence of mood disorder comorbidity in neuropathic pain. Somatostatin-expressing GABAergic neurons in the CeA also project to the PBN. Optogenetic activation of this pathway suppresses acute pain, and its inhibition evokes pain behaviors, demonstrating the pain-inhibitory role of this circuit. Moreover, the inhibitory efficacy of this circuit is suppressed after infraorbital nerve injury, resulting in the hyperexcitability of lateral PBN neurons [63], which further supports the contribution of PBN neurons to neuropathic pain. On the other hand, glutamatergic neurons in the basal lateral amygdala (BLA) innervate neurons expressing corticotropin-releasing factor (CRF) in the CeA. Optogenetic activation of either BLA projections or CRF-positive neurons in the CeA facilitates nociception in rats, while inhibition reduces pain following arthritis [64]. This study identifies a pain-modulatory circuit within the amygdala. In addition, BLA neurons projecting to GABAergic interneurons in the mPFC are activated after sciatic nerve injury, which reduces the activity of layer V pyramidal cells in mice. The contribution to these changes in neuropathic pain has been confirmed by the reversal of mechanical allodynia and thermal hyperalgesia when optically inhibiting this circuit [65]. These studies together demonstrate the key position of the amygdala in pain processing and modulation and its contribution to the comorbidity of pathological pain and mood disorder.

#### Cerebral Cortex

A few subregions of the cerebral cortex such as the somatosensory cortex, insular cortex, and mPFC have been implicated in processing multiple dimensions of pain, including the generation of sensation and affective responses, cognition, and coping. Emerging studies using optogenetics have revealed that the cortex actively participates in pain processing and modulation and contributes to pathological pain through diverse circuits.

The PFC has long been known to participate in encoding the emotional aspects of pain. However, several lines of evidence have demonstrated that the activity profiles of PFC neurons are implicated in pain modulation and chronic pain. The activity of pyramidal neurons in layer V of the PrL is reduced after nerve injury [65–67], possibly owing to the augmented synaptic connections between BLA excitatory neurons and local interneurons [66, 68]. Optogenetic activation of PrL neurons has analgesic and anxiolytic effects in mice with inflammatory pain, while inhibition is anxiogenic in naïve mice [69]. Another study revealed that PrL neurons show increased excitability at the onset followed by reduced excitability in the prolonged phase of neuropathic pain. This switch of neuronal activity as well as behavioral depression in the prolonged phase of pain may be attributed to changes in the dysfunction of endocannabinoid system after nerve injury [70]. In addition, neurons in the infralimbic cortex, a subregion of the mPFC, are normally modulated by the ventral hippocampal CA1, probably *via* BDNF. Persistent spontaneous pain under inflammatory conditions disrupts this modulation, while optogenetic rescue relieves pain, revealing the involvement of this circuit in pain modulation [71]. A few studies have consistently found that optogenetic activation of excitatory neurons in the mPFC attenuates both pain- and anxiety-like behaviors in inflammatory and neuropathic pain models [57, 65, 67, 68], providing evidence that reduced neuronal activity in this region promotes pain hypersensitization and mood disorder comorbidity. This role may be mediated by the weakened output to the vlPAG and the NAc, key targets of the mPFC [57, 67, 68]. Moreover, Liang *et al.* revealed that neuronal nitric oxide synthase-expressing neurons in the mPFC receive excitatory inputs from the posterior subregion of the PVT and transform inflammatory pain signals into anxiety signals by enhancing glutamate transmission [72], emphasizing the role of nitric oxide in the vmPFC in driving the affective comorbidity of chronic pain. In addition, the dysfunction of mPFC neurons has been implicated in memory deficits in neuropathic pain [73, 74]. These studies not only demonstrate the roles of the mPFC in chronic pain and its comorbidities, but also justify the mPFC as a potential therapeutic target.

The orbital cortex has recently attracted interest for its role in neuropathic pain. Glutamatergic neurons in layer V of the ventrolateral orbitofrontal cortex (vlOFC) receive inputs from the ventromedial thalamus (VM) and project to the posterior vlPAG. Activation of this circuit attenuates mechanical and thermal hypersensitivity and cold allodynia in mice after sciatic nerve injury without effect on acute nociception, demonstrating a specific function of the vlOFC–vlPAG circuit in neuropathic hypersensitivity [75]. Another study revealed that the activity of vlOFC glutamatergic neurons is decreased after trigeminal nerve injury. Moreover, specific activation of these neurons blocks anxiodepressive-like behaviors induced by trigeminal neuropathy. However, the anti-anxio-depressive effect is not mediated by the projections to the PAG [76]. The discrepancy between these two studies indicates that not only sensory and affective aspects of neuropathic pain, but also trigeminal and spinal neuropathies may have separate intracerebral mechanisms in terms of the circuits involved.

The ACC is also known for encoding the affective aspect of pain. In support of this, optogenetic activation of ACC neurons is sufficient to induce anxiety and depression-like behaviors in naïve mice [77]. The ACC receives excitatory innervation from S1, and this connection is enhanced in chronic pain states. Modulation of this projection regulates aversive responses to pain, indicating the role of this circuit in linking sensory and affective pain signals [78]. Moreover, the firing rate and bursting activity of ACC neurons increase when anxiodepressive-like behaviors develop after nerve injury and the hyperactivity persists after the hypersensitivity recovers [79]. The findings that ACC lesions prevent the anxiodepressive consequences of neuropathic pain without affecting allodynia [77] and that optogenetic inhibition of ACC neurons alleviates the aversive and anxiodepressive-like consequences of neuropathic pain [79] together demonstrate that the ACC hyperactivity is essential for driving the emotional impact of neuropathic pain. In line with this notion, activating glutamatergic inputs from the mediodorsal thalamus elicits pain-related aversion in neuropathic pain models [80]. On the other hand, accumulating evidence suggests that the ACC is also involved in pain modulation. Selective activation of inhibitory neurons in the ACC inhibits pain and the responses of pyramidal neurons to painful stimuli [81, 82], while activation of glutamatergic neurons facilitates nociception [82, 83]. In addition, inhibition of ACC excitatory neurons is able to reduce cancer pain [84]. Activation of ACC neurons expressing D1 markedly exacerbates the trigeminal neuropathic pain induced by infraorbital nerve injury, while activation of D2-expressing neurons has an analgesic effect, demonstrating the diverse roles of ACC neurons in modulating trigeminal neuropathic pain [85]. Studies have revealed that the pain-modulatory action of ACC neurons may be mediated by descending projections. Optogenetic inhibition of the ACC decreases the discharges of sensory thalamic neurons [86, 87], while activation or inhibition of the ACC–dorsomedial striatum circuit enhances or attenuates nociception and neuropathic pain [88], both indicating that the thalamus and striatum are involved in the pain-modulating circuitry of the ACC. In addition, activation of neurons projecting to the spinal cord potentiates spinal excitatory transmission and causes behavioral hypersensitivity, while inhibiting these projections has an analgesic effect [35], demonstrating that besides the subcortical brain regions, the spinal cord is modulated directly by the ACC. Interestingly, a recent study has reported that selective inhibition or activation of the ACC–NAc circuit impairs or enhances the social transfer of pain and analgesia, rather than fear [89], revealing the selective involvement of this circuit in empathy for pain.

The midcingulate cortex (MCC) is no longer considered to be the caudal part of the ACC, but is now seen as an independent entity [90]. Human brain imaging studies have identified the MCC as a generalizable representation of acute nociceptive pain [91]. Tan *et al.* optogenetically manipulated MCC glutamatergic neurons and found that they do not mediate acute pain perception, neuropathic allodynia, and the associated negative affect, but gate capsaicin-induced sensory hypersensitivity through a circuit to the posterior insula [92]. However, we later found that optogenetic activation of glutamatergic neurons in the dorsal (Cg1) and ventral (Cg2) parts of the MCC has algesic and analgesic effects on mechanical nociception and neuropathic allodynia, respectively. Neurons in these two subregions exhibit distinct changes in activity that favor pain sensitization after nerve injury. We also found that the analgesic effect of Cg2 is largely mediated by projections to GABAergic neurons in the zona incerta [93]. Therefore, our study distinguishes the opposite roles of MCC neurons in two subregions in pain. The discrepancies between these two studies may be explained by the differences in the connections, neuronal composition, and functions in the Cg1 and Cg2 subregions of the MCC.

The sensorimotor cortex not only controls the sensation of and responses to afferent pain signals, but also sends dense efferents to other brain regions that integrate and modulate these signals. For example, S1 relays noxious signals to the ACC to elicit aversive responses [78]. Optogenetic activation of excitatory S1 neurons at beta and gamma frequencies blocks the transmission of pain signals [94], whereas gamma oscillations induced by optogenetic activation of PV-expressing GABAergic neurons enhances nociception and induces aversion [95]. Nerve injury and persistent inflammatory pain induce hyperexcitability of neurons in S1 layer V. Optogenetic rescue of their activity relieves pain-like behaviors and comorbid anxiety [96, 97], at least in part *via* projections to GABAergic neurons in the caudal dorsolateral striatum and the caudal part of the spinal trigeminal nucleus [96, 98]. These studies provide direct evidence for the involvement of S1 in pain processing and pathological pain. In addition, optical activation of the primary motor cortex inhibits neuronal discharge in the sensory thalamus, and alleviates trigeminal and spinal neuropathic pain in rats [99, 100], replicating the analgesic effect of motor cortex stimulation. These studies justify the application of non-invasive stimulation approaches that are capable of modulating the sensorimotor cortex for pain management.

## Summary and Perspective

In summary, the application of optogenetics increases the depth and diversity of pain studies. The findings support the current knowledge that a complex network is involved in both pain transmission and modulation from the periphery to the cortex, yet none of the components in this network has been demonstrated to be specifically linked to pain. Most studies reveal new circuits for pain, while some confirm or support previous studies. In the periphery, both types Aβ and C afferent fibers, including peptidergic and non-peptidergic, participate in pain transmission and pathological pain. At the spinal level, modulation of relay neurons derives from local and supraspinal structures, even directly from the cortex. Excitatory or inhibitory modulation occurs both at presynaptic and postsynaptic sites, supporting and expanding the pain gate control theory. In the brain, a more complex circuitry for pain processing is built by a variety of nuclei. Although many of them have been implicated in pain such as the PAG, PBN, ACC, and amygdala, optogenetics enables more direct, precise, and delicate studies on molecule-dependent circuits and the neurotransmitters involved. Dysfunction of any components in this network can lead to pathological pain, highlighting the complexity of its pathogenesis. A specific circuit may participate in modulating nociception but not pathological pain, and *vice versa*. Importantly, it is very likely that different sets of circuits are involved in different aspects of pain, especially in chronic pain and its affective consequences. Deconstruction of the cellular, molecular, and circuit mechanisms of acute and chronic pain will certainly pave avenues to new effective pharmacological and/or nonpharmacological strategies for pain management. The findings from optogenetics justify the development and application of multidisciplinary approaches or versatile targets for the management of pain. In addition, the interactions between pain and other brain functions such as affective states, are elusive. Optogenetics can serve as a useful tool to answer these questions.

However, the volume of most brain nuclei is much larger than the area that can be illuminated by light. Moreover, many nuclei have complex heterogeneity in cellular composition and distribution topology. It is no wonder that the results from different studies on the same nucleus may be conflicting when the methodologies are different. Detailed characterization of molecular markers and the distribution of cells in certain brain areas is crucial for improving the specificity of optogenetic manipulation and evoked biological effects. In addition, it is technically very difficult to simulate the intrinsic discharge patterns using optical stimulation, especially activation approaches. Thus, caution should be taken in interpreting the results of optical manipulation. Along with the advancements in cell biology and optogenetic technology, we hope that these limitations will be gradually overcome.
